# Diagnostic performance of the rapid test for the detection of NS1 antigen and IgM and IgG anti-antibodies against dengue virus

**DOI:** 10.17843/rpmesp.2022.394.11471

**Published:** 2022-12-23

**Authors:** Begoña Valdivia-Conroy, Jefferson M. Vasquez-Calderón, Wilmer Silva-Caso, Johanna Martins-Luna, Miguel Angel Aguilar-Luis, Juana del Valle-Mendoza, Zully M. Puyén

**Affiliations:** 1 Universidad Peruana de Ciencias Aplicadas, Lima, Peru. Universidad Peruana de Ciencias Aplicadas Universidad Peruana de Ciencias Aplicadas Lima Peru; 2 Instituto Nacional de Salud, Lima, Peru. Instituto Nacional de Salud Lima Peru

**Keywords:** Dengue, NS1 Antigen, IgM, IgG, Rapid Test, Diagnosis, Peru, DENV

## Abstract

**Objectives:**

. To assess the diagnostic performance of the SD dengue DUO rapid test (Inyecta) for the detection of NS1, IgM and IgG in comparison to the ELISA test.

**Materials and methods:**

. This is a diagnostic test evaluation that included 286 serum samples from patients with symptomatology attributable to dengue from endemic areas of Peru. The samples were analyzed by ELISA and the SD dengue DUO rapid test (Inyecta) for IgM, NS1 and IgG at the Instituto de Investigación Nutricional in Lima.

**Results:**

. The sensitivity of the rapid test was 68.0% for NS1 and IgM, and 86.0% for IgG, improving to 75.0% and 81.0% for NS1 and IgM, respectively, during the first three days. The specificity for all three analytes was greater than 87.0%. The concordance of the results, measured by the Kappa coefficient for the three analytes, was good and no cross-reaction with other arboviruses was found.

**Conclusions:**

. The SD dengue DUO rapid test allows detection of NS1, IgM and IgG with adequate sensitivity and specificity. Sensitivity for IgM and NS1 increases when detected during the first three days of symptoms. Therefore, we recommend its implementation in primary care centers for early and timely diagnosis.

## INTRODUCTION

Dengue is a disease caused by a virus of the Flaviviridae family called dengue virus (DENV) and can be caused by any of the four known serotypes (DENV-1, DENV-2, DENV-3 and DENV4). The virus is transmitted by mosquitoes of the Aedes genus and usually exists in tropical and subtropical climates, in urban or semi-urban areas ^1^. This disease is a major public health problem worldwide, and the American region is not exempt from this problem. It currently affects different Latin American countries, with Brazil reporting the highest number of cases (1,040,481), followed by Paraguay (218,696 cases), Argentina (72,701 cases), Bolivia (82,460 cases), Colombia (54,192 cases) and Peru (26,543 cases) ^2^^,^^3^. In Peru, dengue is a growing major public health problem due to different factors including: the increase in mosquito resistance to insecticides, the increase in disorganized urbanization, the lack of drinking water and the habit of keeping water in uncovered containers for reasons such as lack of knowledge or poor waste collection practices ^3^.

Initial diagnosis is clinical, followed by laboratory tests. At the onset of the disease, clinical manifestations include a wide spectrum of symptoms ranging from asymptomatic or mild infection and typical dengue fever to hemorrhagic fever and dengue shock syndrome. On the other hand, this disease can be diagnosed by direct laboratory tests (viral isolation by culture and detection of nucleic acids by reverse transcriptase PCR [RT-PCR]) or indirect tests (detection of IgM and IgG antibodies by ELISA). Direct tests can be performed by conventional RT-PCR and real-time RT-PCR (qRT-PCR), including the detection of the dengue non-structural glycoprotein 1 (NS1) antigen, which is secreted by infected cells as a soluble hexamer. Indirect tests can detect antibodies, either IgM and IgG through enzyme-linked immunosorbent assays (ELISA) ^4^. Each of these tests has been previously evaluated and implemented in laboratories of different levels in Peru, however, they are expensive, require a complex infrastructure and in some cases, high levels of biosafety measures, in addition to requiring specialized equipment and trained personnel ^5^.

In contrast to what was previously mentioned, rapid immunochromatographic tests (also called lateral flow tests) are an alternative that can be implemented in limited resource scenarios, becoming a rapid and timely strategy for the *in situ* evaluation of viral infection and antibodies, additionally supporting the epidemiological surveillance of the disease in endemic areas. However, these tests have shown low sensitivity due to cross-reaction with other viruses of the flavivirus family, so they usually have a low negative predictive value ^6^.

Currently, there is a wide availability of rapid tests that offer better performance results for the evaluation and approach to diagnosis, so evaluating them is an important strategy for searching new high-performance diagnostic alternatives.

In Peru, the criteria for a probable case of dengue without warning signs include fever less than or equal to one week, residing in or having visited areas of transmission 14 days before the onset of symptoms and presenting at least two of the following symptoms: ocular or retro ocular pain, myalgia, headache, arthralgia, back pain, skin rash, nausea and vomiting. Likewise, a probable case with alarm signs refers to the aforementioned criteria with the addition of one or more of the following: severe abdominal pain, chest pain, dyspnea, hypothermia, spontaneous mucosal bleeding, Glasgow score less than 15, hepatomegaly, hemogram compatible with hemoconcentration (hematocrit and hemoglobin greater than the expected range or the patient’s previous figures). On the other hand, a case of severe dengue meets the characteristics of a probable case associated with hypovolemic shock, hemorrhage, pulmonary edema or significant organ involvement. Finally, a confirmed case of dengue is a probable case with positive RT-PCR, NS1 antigen ELISA, IgM ELISA in endemic areas, IgM seroconversion in paired samples, or virus isolation in culture ^6^.

In general, qRT-PCR tests are performed in patients with one to five days of illness and allow the detection of the nucleic acid of the virus; they are highly sensitive and specific tests ^7^. NS1 antigen can be detected from onset to day 18 by lateral flow rapid test, by immunochromatographic assay or by ELISA ^8^. IgM can be detected four days after the onset of fever by ELISA or lateral flow rapid test ^6^, the presence of IgG indicates a secondary or past infection.

The rapid test is used during the acute phase of dengue infection. However, its diagnostic sensitivity could be negatively affected in endemic areas, as secondary flavivirus infections are common ^9^. In low-socioeconomic settings with limited access to healthcare centers, rapid tests often become the only option for patient diagnosis; they are really important in these scenarios for the early and timely identification of outbreaks, avoiding procedures that require more training, biosafety measures, equipment and complex infrastructure ^9^. Therefore, the present study focused on providing support to endemic regions that do not have equipment or trained personnel to perform sophisticated diagnostic tests at the time of patient recruitment. In this way, public health policies on epidemiological surveillance, early diagnosis and timely treatment can be strengthened.

Therefore, the aim of this study was to determine the diagnostic performance of a rapid serological test for the detection of NS1 antigen and IgM and IgG antibodies against dengue virus from serum samples of Peruvian patients with symptoms attributable to dengue. For this purpose, we sought to establish the sensitivity and specificity of the rapid test for the detection of NS1, IgM and IgG, using indirect ELISA as the reference test for each of the three analytes separately. ELISA is used as the reference test because it allows the identification of both antigen and antibodies and is the one implemented in the country.

**KEY MESSAGES**

Motivation for the study: search for accessible and efficient new diagnostic alternatives for the detection of the disease caused by the dengue virus.

Main findings: good efficiency of the rapid test during the first days of the disease. As well as its high power to discriminate against other similar mosquito-borne diseases such as Zika and Oropuche.

Implications:it could be applied as a screening test in endemic regions that do not have equipment or trained personnel to perform sophisticated and/or complex diagnostic tests. Strengthening public health policies in epidemiological surveillance, early diagnosis and timely treatment.

## MATERIALS AND METHODS

This is a diagnostic-test observational study that used serum samples collected from patients with symptoms attributable to dengue during the years 2019-2020, within the framework of the Epidemiological Surveillance Program. The samples were stored at -80 °C in the sample bank of the Institute of Nutritional Research. These samples were positive or negative for dengue virus infection by the Euroimmun ELISA test, for NS1, IgM and IgG separately. All the processes involved in the assessment of the tests were carried out blindly by the analysts. We considered the STARD guidelines for diagnostic test studies for the writing of this manuscript.

Regarding sample calculation, we used the study by Vásquez *et al*. in 2012 ^10^^)^ as reference, which showed the test they analyzed reached a general sensitivity of 93.8% and a specificity of 95.8%. By considering a precision of 5.0%, we obtained a minimum sample size of 152, 90 would be positive and 62 would be negative by ELISA. The present study analyzed a total of 286 serum samples, of which 203 were positive and 83 were negative by ELISA.

Diagnostic performance was determined by calculating the parameters of sensitivity, specificity, positive and negative predictive value, as well as Cohen’s Kappa coefficient and the likelihood ratio (LR) of a rapid test that detects NS1, IgM and IgG analytes against dengue virus called SD Dengue Duo-Inyecta, compared with the results obtained by the Euroimmun ELISA test. Additionally, one of the variables we included was the symptomatic period, which was measured from the onset of symptoms attributable to dengue virus and was divided as follows: 1-3 days, 4-7 days and 8-14 days. Specifically, NS1 appears in the first three days and disappears by day 9, IgM appears 3-5 days after symptom onset and persists for approximately two weeks. Finally, IgG appears from day 10, when the infection is primary; however, if it is secondary, it could be detected even on day 4 and can remain high for several months ^5^. Other variables, such as age and sex, were also evaluated.

Finally, as an additional step, 42 samples that tested positive for Oropuche virus _^(22)^_ , Zika virus ^8^ and Chikungunya virus ^12^ were included among the negative samples, in order to evaluate cross-reactivity. All statistical analyses were carried out with Stata 16.0 (College Station, TX) and OpenEpi version 3.

### SD Dengue DUO® Rapid Test (INYECTA)

The SD Dengue Duo® Rapid Test (product 11FK45-P-2, lot 11DDC007A, STANDARD DIAGNOSTIC, INC. Germany) is an immunochromatographic test that detects NS1 antigen and IgM/IgG antibodies in a patient’s blood sample ^11^. The rapid test has two compartments, one for NS1 and the other for IgM/IgG with a control line that should always be marked to ensure the validity of the result. The left compartment is for NS1 antigen where three drops of serum are added, and the right compartment is for IgM/IgG where 10 µl of serum and four drops of diluent are added. Results are obtained in 20 min. A positive NS1 result is considered if there are two marked lines. A positive IgM result is considered if the control line and the line indicating IgM are marked. A positive IgG result is considered when the control line and the IgG line are marked. The whole procedure was conducted according to the manufacturer’s stipulations ^12^.

### Indirect ELISA test

This test requires a minimum of 2 ml of blood. Once the sample is collected, it has to be stored at 2-8 °C until sent to the laboratory; results can be obtained within a maximum of three days. 

DENV NS1 antigen and IgM/IgG antibodies were detected using Euroimmun ELISA (Euroimmun AG, Lübeck, Germany) according to the manufacturer’s instructions. For each case: DENV NS1 ELISA (lot E21102020DU, cat EQ 266a-9601-1); DENGUE IgM ELISA (lot E211202CA, cat EI 266a-9601-1M) and DENGUE IgG ELISA (lot E210312AF, cat EI 266a-9601-1G) were used.

### Ethical Aspects

Authorization was requested from the ethics committee of the Universidad Peruana de Ciencias Aplicadas in order to carry out this research. After being accepted with document FCS-CEI/281-08-20, we requested the database from the Instituto de Investigación Nutricional. 

This study did not involve risks for the patient, since the researchers only collected information from the samples obtained by the Epidemiological Surveillance Program and processed by the National Institute of Nutrition. In addition, all the information collected was confidential, double coded, and could only be accessed by the researchers of this study. Finally, this research did not require informed consent because the participant was not contacted; we analyzed samples that had previously been collected and processed.

## RESULTS

A total of 286 samples were evaluated for NS1, IgM and IgG using the new SD Dengue DUO-Inyecta rapid test and the Euroimmun ELISA reference test. Overall, the positivity rates for NS1, IgM and IgG by the rapid test were 24.5% (70), 13.0% (37) and 52.5% (150), respectively; meanwhile, the positivity rates for the reference test were 34.0% (97), 19.0% (54) and 54.5% (156), respectively. Additionally, 60.0% of patients were between day 1 and 3 since symptom onset (Table 1).

**Table 1:** Clinical-demographic characteristics and results of patients with symptoms attributable to dengue included in the study.

Variables	n	%
Sex		
Men	128	55.2
Women	158	44.8
Age (years)	32.8 (2-80) ^a^	
Symptomatic period		
1-3 days	172	60.2
4-7 days	95	33.2
8-14 days	11	3.8
≥15 days	8	2.8
ELISA IgM		
Positive	54	19.0
Negative	232	81.0
ELISA IgG		
Positive	156	54.5
Negative	130	45.5
ELISA NS1		
Positive	97	34.0
Negative	189	66.0
SD dengue IgM		
Positive	37	13.0
Negative	249	87.0
SD dengue IgG		
Positive	150	52.5
Negative	136	47.5
SD dengue NS1		
Positive	70	24.5
Negative	216	75.5

a Median, age range.

IgM: immunoglobulin M, IgG: immunoglobulin G, NS1: nonstructural protein, ELISA: enzyme-linked immunosorbent assay.

Results of the SD dengue DUO-Inyecta rapid test and the Euroimmun ELISA for detecting NS1, IgM and IgG from serum samples show false positive and negative results for the three analytes, except for IgM, which showed no false positives (Table 2). Table 3 shows the performance of the rapid test for detecting the three analytes. Sensitivity was around 68.0% for NS1 and IgM and was 20.0% higher for IgG; specificity and positive predictive value of the rapid test were generally higher than 87.0%.

**Table 2:** Comparison of results obtained for NS1, IgM and IgG by the SD Dengue DUO-Inyecta rapid test and Euroimmun ELISA from serum samples of patients with dengue-attributable symptomatology.

SD Dengue DUO-Inyecta rapid test positive		Euroimmun ELISA		
		Positive	Negative	Total
NS1	Positive	65	5	70
	Negative	32	184	216
	Total	97	189	286
IgM	Positive	37	0	37
	Negative	17	232	249
	Total	54	232	286
IgG	Positive	134	16	150
	Negative	22	114	136
	Total	156	130	286

IgM: immunoglobulin M, IgG: immunoglobulin G, NS1: non-structural protein; ELISA: enzyme-linked immunoadsorption assay.

**Table 3:** Diagnostic performance of the SD Dengue DUO-Inyecta rapid test compared to Euroimmun ELISA for the detection of NS1, IgM, IgG in serum samples from patients with Dengue-attributable symptomatology.

	NS1 (95% CI)	IgM (95% CI)	IgG (95% CI)
Sensitivity %	67.0	68.5	85.9
	(57.2-75.6)	(55.3 - 79.3)	(79.5 - 90.5)
Specificity %	97.4	100	87.7
	(94.0 - 99.0)	(98.4 - 100.0)	(81.0 - 92.3)
PPV %	92.9	100.0	89.3
	(84.3 - 96.9)	(90.6 - 100)	(83.4 - 93.3)
NPV %	85.2	93.2	83.8
	(79.8 - 89.3)	(89.3 - 95.7)	(76.7 - 89.1)
Cohen’s Kappa	0.69	0.78	0.73
	(0.57 - 0.80)	(0.67 - 0.89)	(0.62 - 0.85)
Positive LR	25.33	68.52 ^a^	6.979
Negative LR	0.33	0.31	0.16

NS1: nonstructural protein, IgM: immunoglobulin M, IgG: immunoglobulin G, PPV: positive predictive value, NPV: negative predictive value, 95% CI: 95% confidence interval, LR: likelihood ratio, +LR: likelihood ratio. ^a^ A specificity of 99% was used to find +LR.

Negative predictive values were 83.0% or higher. The κ coefficients of all comparisons were at 0.69 or higher; for IgM it was 0.78. In addition, the positive likelihood ratio (LR) for NS1 antigen and IgM antibody were greater than 10; it was 6.979 for IgG. On the other hand, the negative LR was similar for NS1 (0.33) and IgM (0.31), but lower for IgG with a value of 0.16. Additionally, we found that the sensitivity of the rapid test increased for the three analytes during the first three days of symptoms, however, as the days go by, the sensitivity decreases (Table 4).

**Table 4:** Sensitivity of the SD Dengue DUO-Inyecta rapid test compared to the Euroimmun ELISA for the detection of NS1, IgM, IgG according to the number of days after the onset of symptomatology.

	Days after symptom onset		
SD Dengue DUO-Inyecta	1-3 days (95% CI)	4-7 days (95% CI)	8-14 days (95% CI)
NS1	75,3%	61.9%	50.0%
	(64.4 - 83.8)	(40.9 - 79.3)	(9.5 - 90.5)
IgM	80.8%	57.7%	100%
	(62.1 - 91.5)	(39.0 - 74.5)	(20.7 - 100.0)
IgG	99.0%	91.5%	85.7%
	(94.5 - 99.9)	(80.1 - 96.7)	(48.7 - 97.4)

IgM: immunoglobulin M, IgG: immunoglobulin G, NS1: nonstructural protein 1, 95% CI: 95% confidence interval

Finally, the cross-reactivity (analytical specificity) of the SD dengue DUO-Inyecta rapid test with different viruses such as Oropuche, Zika and Chikungunya was also evaluated, obtaining a result for this parameter of 100.0%, which means no cross-reactivity of the rapid test was found with the selected viruses.

## DISCUSSION

The SD Dengue Duo-Inyecta serological rapid test showed a sensitivity of over 67.0% and a specificity of over 97.0% for NS1 and for IgM, with sensitivity increasing to 75.0% for NS1 when applied within the first three days of symptom onset. Very similar results were found in the study by Chung-Hao *et al.*^9^. Likewise, it is emphasized that one of the advantages of this test is that it allows early detection of the disease, especially during its acute phase.

Previous studies have assessed rapid tests used for the detection of analytes against dengue, most of them have been compared with more complex diagnostic tests (requiring elaborate infrastructures, highly trained personnel and specialized equipment). Thus, in 2012, the SD Bioline Dengue Duo diagnostic test was compared with a standard diagnostic test called Platelia NS1, obtaining a sensitivity of 57.8% and a specificity of 98.0% ^10^. Likewise, another study evaluated the Dengue NS1 Ag STRIP rapid test and found a sensitivity of 63.8% in 392 patients with confirmed dengue diagnosis ^9^. Additionally, in Colombia, a study analyzed the diagnostic effectiveness of the “Dengue NS1 and IgM rapid test” during a pre-Zika period in 2019, reporting a clinical diagnostic sensitivity of 61.4% ^11^. On the other hand, a study carried out in Lambayeque, Peru, in 2013, also analyzed the diagnostic effectiveness of the SD Bioline Dengue Duo rapid test compared to the ELISA test, and reported a sensitivity of 88.5% ^13^.

Our results show a sensitivity of 67.0% and 68.5%, and a specificity of 97.4% and 100.0% for NS1 and IgM, respectively. The sensitivity and specificity results obtained for IgG are around 86.0%, and this may be due to the presence of a significant percentage of secondary infections, so these results should be interpreted with caution. These data are similar to the results reported by Clemen *et al*. in 2019 ^11^ as well as to a study that evaluated the Dengue NS1 Ag STRIP rapid test ^9^. In addition, the sensitivity obtained for IgM and NS1 in our study was higher than that found in other articles that also evaluated the diagnostic performance of different rapid tests, this could be due to the fact that the different DENV serotypes were not analyzed; if the DENV serotype 2 and 4 would predominate, then the sensitivity of the NS1 antigen would have been altered. However, sensitivity was higher in the research carried out in Peru in 2013 ^13^, perhaps this increase in sensitivity of some tests may be due to the fact that the symptomatic period of the disease was not taken into account, thus increasing sensitivity. Likewise, other studies evaluated the sensitivity and specificity of different rapid tests, obtaining similar results ^10^^,^^14^^-^^15^.

On the other hand, in 2019 ^11^, a research sought to compare the diagnostic efficacy of the SD dengue DUO rapid test with real-time PCR (gold standard). That study resulted in a sensitivity of 85.4% and specificity of 94.5% for the rapid test, whereas the present study showed lower sensitivity for NS1 and IgM and similar specificity for NS1 and IgM, but lower for IgG. This difference between both studies may be caused by the reference test used, since the first study was based on PCR and ours used indirect ELISA. The latter in order to replace the test that is massively implemented by MINSA in the regions of Peru, which is the ELISA.

In our research, more than 50.0% of the included population were positive for IgG antibodies, suggesting a secondary infection, which also causes an independent factor for a negative NS1 result. It has been suggested that this could be due to the similarity between the kinetics of specific NS1-IgG and DENV IgG, which causes the formation of immune complexes by the union of the NS1 antigen and specific NS1-IgG, which is an antibody that appears after eight days of a primary infection, but appears on the first day of a secondary infection, which reduces its sensitivity ^16^. Likewise, when evaluating the predictive values for each of the studied analytes, it is important to bear in mind that the results must be taken with caution, since this parameter depends on the prevalence of the disease, therefore these results can only be extrapolated to places where the prevalence is similar, considering the analyte.

Additionally, Cohen’s Kappa coefficient shows that the chance of having a negative result by the rapid test, when positive by ELISA, is low. Similarly, the possibility of having a positive result by the rapid test when it is negative by ELISA is higher than that mentioned above. This is similar to what was reported by Sáenz Bolaños *et al*. in the evaluation of the rapid test against MAC-ELISA in Costa Rica ^17^, which may be due to the similarity of that study with ours and the usefulness of the rapid test during the acute phase of the disease for detecting IgM antibodies.

Similarly, regarding the LR obtained by the present study, NS1 and IgM showed a highly relevant LR +, since it is 25.33 and 68.52 times more likely that a patient with positive ELISA obtains a positive rapid test for dengue than a patient with negative ELISA has the same result; a good utility was also found for IgG (6.79). As for negative LR, a useful value was found for IgG (0.16), but the other analytes showed regular results (0.31), so it can be affirmed that the negative results of the rapid test occur 0.31 times in patients with positive ELISA with respect to each sample with negative ELISA. Additionally, the interpretation of these results has an important role, especially when defining the prevalence of the disease. By considering different scenarios of disease prevalence (low, medium or high) and with the LR results obtained by means of the test, the clinical interpretation that this parameter can provide could be better evaluated ^18^.

Additionally, we found that the diagnostic performance of the rapid test changes according to the symptomatic period of the patient and the type of analyte. In our study, the detection of IgM reached a sensitivity of 80.77% during the first three days of illness, from the onset of symptoms. This also occurs with the NS1 antigen that reached its highest point of sensitivity during the first three days, 75.3%. This confirms its usefulness during the acute phase of the disease and that as the days pass, sensitivity decreases. However, the high sensitivity during the first days for the detection of IgG is an indicator of reinfection by the virus.

On the other hand, when evaluating the analytical specificity of the SD Dengue Duo-Inyecta rapid test with respect to other arboviruses such as Zika, Chikinguya and Oropuche, the rapid test showed a specificity of 100.0% for dengue, which indicates that there is no cross-reactivity with other arboviruses, validating its usefulness in endemic areas for these diseases. Cross-reactivity with the Mayaro virus was not evaluated, despite the fact that its distribution has increased in Peru ^19^.

The efficiency of the rapid test during the first days of the disease is one of our main findings, as well as its capacity to distinguish dengue disease from similar ones such as Zika or Oropuche, which are also transmitted by mosquitoes. One of the limitations of our study is that the clinical-epidemiological information was scarce, because the samples had been collected for a different purpose and were stored in the laboratory’s sample bank. Likewise, not using PCR, which is considered the gold standard test for dengue diagnosis, limited the assessment of the sensitivity and specificity of the rapid test when compared to the molecular test; however, the aim of our study included replacing the ELISA test, which is the one implemented in different regions of the country. It is also important to mention that the information about the symptomatic period was provided by the patient, which could have produced memory bias by incorrectly giving the date of symptom onset. Finally, not knowing the dengue serotype or whether the infection was primary or secondary could alter the performance of the rapid test evaluated.

In conclusion, the diagnostic performance of the SD Dengue Duo-Inyecta rapid serological test for the detection of NS1 antigen and IgM and IgG antibodies indicates that it is an acceptable method for early dengue diagnosis. Also, our results should be treated with caution in patients with more than seven days from the onset of symptoms, as the sensitivity of the test may be affected.

We recommend the implementation of this test in primary health care facilities in endemic areas in order to achieve a rapid and timely diagnosis, reducing the transmission and complication of the disease, as well as facilitating the early detection of possible outbreaks. It is important to note that the rapid test provides a useful presumptive diagnosis but does not give a definitive diagnosis, so another test such as ELISA or PCR should also be performed to confirm the diagnosis.
