# Supplementary material for: Diagnostic performance of the rapid test for the detection of NS1 antigen and IgM and IgG anti-antibodies against dengue virus
Source: Rev Peru Med Exp Salud Publica. 2022 Dec 23;39(4):434–41. doi: 10.17843/rpmesp.2022.394.11471 (PMC11397755; doi:10.17843/rpmesp.2022.394.11471)
Supplement: Translation

# Translation: Rendimiento diagnóstico de la prueba rápida para la detección del antígeno NS1 y anticuerpos IgM e IgG contra el virus del dengue

**Authors:** Begoña Valdivia-Conroy, Jefferson M. Vasquez-Calderón, Wilmer Silva-Caso, Johanna Martins-Luna, Miguel Angel Aguilar-Luis, Juana del Valle-Mendoza, Zully M. Puyén

**Affiliations:** 1 Universidad Peruana de Ciencias Aplicadas, Lima, Perú.; 2 Instituto Nacional de Salud, Lima, Perú.

**Keywords:** Dengue, Antígeno NS1, IgM, IgG, Prueba Rápida, Perú, Diagnóstico, DENV

## Abstract

**Objetivos.:**

Determinar el rendimiento diagnóstico de la prueba rápida SD dengue DUO (Inyecta) para la detección de NS1, IgM e IgG en comparación con la prueba de ELISA.

**Materiales y métodos.:**

Es una evaluación de prueba diagnóstica que incluyó 286 muestras de suero de pacientes con sintomatología atribuible a dengue de zonas endémicas del Perú. Las muestras se analizaron por ELISA y la prueba rápida SD dengue DUO (Inyecta) para IgM, NS1 e IgG en el Instituto de Investigación Nutricional en Lima.

**Resultados.:**

La sensibilidad de la prueba rápida fue de 68% para NS1 e IgM, y 86% para IgG, mejorando este parámetro a 75% y 81% para NS1 e IgM, respectivamente, en los tres primeros días. La especificidad para los tres analitos fue mayor a 87%. La concordancia de los resultados obtenidos medidos por el coeficiente *Kappa* para los tres analitos fue buena y no se encontró reacción cruzada con otros arbovirus.

**Conclusiones.:**

La prueba rápida SD Dengue DUO permite detectar con una adecuada sensibilidad y especificidad NS1, IgM e IgG. La sensibilidad para IgM y NS1 aumenta cuando se detecta en los tres primeros días de síntomas, por lo que se recomienda su implementación en los centros de primer nivel de atención para un diagnóstico temprano y oportuno.

## INTRODUCCIÓN

El dengue es una enfermedad causada por un virus de la familia Flaviviridae denominado el virus del dengue (DENV) y puede ser causada por cualquiera de los cuatro serotipos conocidos (DENV-1, DENV-2, DENV-3 y DENV4). El virus es transmitido por los mosquitos del género Aedes el cual suele presentarse en zonas donde el clima es tropical y subtropical, pudiendo ser en áreas urbanas o semiurbanas ^1^. En el mundo, esta enfermedad es un gran problema de salud pública, y la región América no se ve exenta de este problema, es por esto que en la actualidad esta patología afecta de forma importante a diferentes países, siendo Brasil el que reporta la mayor cantidad de casos (1 040 481), seguidos por Paraguay (218 696 casos), Argentina (72 701 casos), Bolivia (82 460 casos), Colombia (54 192 casos) y Perú con 26 543 casos ^2^^,^^3^. En el Perú el dengue es uno de los principales problemas de salud pública que va en crecimiento debido a diferentes factores, entre ellos: el aumento de la resistencia del mosquito a insecticidas, el incremento de la urbanización desorganizada acompañado de la falta de agua potable y el almacenamiento del agua en recipientes descubiertos por razones como la falta de conocimiento y deficiente recolección de residuos ^3^.

El diagnóstico inicial es clínico, seguido por el diagnóstico laboratorial. Al inicio de la enfermedad las manifestaciones clínicas incluyen un amplio espectro de síntomas que van desde una infección asintomática o leve y fiebre característica del dengue, hasta fiebre hemorrágica y síndrome del shock del dengue. Por otro lado, en el laboratorio se puede diagnosticar esta enfermedad mediante metodologías directas (aislamiento viral por cultivo y detección de ácidos nucleicos por transcriptasa inversa por PCR [RT-PCR]) o indirectas (detección de anticuerpos IgM e IgG por ELISA). Para el caso de las metodologías directas, estas pueden realizarse mediante RT-PCR convencional y RT-PCR a tiempo real (qRT-PCR), incluyéndose en este caso la detección del antígeno de la glucoproteína no estructural 1 (NS1) del dengue la cual es secretada por las células infectadas como un hexámero soluble. Para el caso de las metodologías indirectas se puede evaluar la detección de anticuerpos, ya sea IgM e IgG a través de pruebas inmunoenzimáticas (ELISA) ^4^. Cada una de estas pruebas han sido previamente evaluadas e implementadas en diferentes niveles de laboratorios en el Perú, sin embargo, estas resultan costosas, requieren de una infraestructura compleja y en algunos casos altos niveles de bioseguridad, además de requerir equipamiento especializado, así como personal capacitado ^5^.

En contraposición a lo mencionado previamente, las pruebas inmunocromatográficas rápidas (también llamadas de flujo lateral) son una alternativa que permite su implementación en escenarios de recursos limitados, tornándose en una estrategia rápida y oportuna para la evaluación *in situ* de la infección viral, así como de los anticuerpos que se generan, apoyando adicionalmente en la vigilancia epidemiológica de la enfermedad en áreas endémicas. Sin embargo, estas pruebas han demostrado baja sensibilidad debido a la reacción cruzada con otros virus de la familia de los flavivirus, por lo que suelen tener un valor predictivo negativo bajo ^6^^).^

Actualmente, existe una amplia disponibilidad de pruebas rápidas que ofrecen mejores resultados de rendimientos para la evaluación y acercamiento al diagnóstico, por lo que las evaluaciones que se hagan de las mismas constituyen una estrategia importante para la búsqueda de nuevas alternativas diagnósticas y de alto rendimiento.

En el Perú, para definir un caso probable de dengue sin signos de alarma, la persona debe cumplir con ciertos criterios, como son: fiebre menor o igual a una semana, que resida o ha visitado áreas de transmisión, 14 días antes del inicio de los síntomas y que presenta al menos dos de los siguientes síntomas: dolor ocular o retro ocular, mialgias, cefalea, artralgias, dolor lumbar, erupción cutánea, náuseas y vómitos. Así mismo, un caso probable con signos de alarma hace referencia a los criterios antes mencionados con adición de uno o más de las siguientes manifestaciones: dolor abdominal intenso, dolor torácico, disnea, hipotermia, sangrado espontaneo de mucosas, Glasgow menor a 15, hepatomegalia, hemograma compatible con hemoconcentración (hematocrito y hemoglobina mayor al rango esperado o a valores previos del paciente). Por otro lado, un caso de dengue grave cumple con las características de un caso probable acompañado de shock hipovolémico, hemorragia, edema pulmonar o compromiso importante de órganos. Finalmente, un caso confirmado de dengue es un caso probable con resultado positivo a RT-PCR, ELISA antígeno NS1, ELISA IgM en zonas endémicas, seroconversión IgM en muestras pareadas, o aislamiento del virus en cultivo ^6^.

De manera general, las pruebas de qRT-PCR se procesan en pacientes con uno a cinco días de enfermedad y permiten detectar la presencia del ácido nucleico del virus, son pruebas altamente sensibles y específicas ^7^. El antígeno NS1 puede detectarse desde el inicio hasta el día 18 mediante las pruebas rápidas de flujo lateral (*lateral flow rapid test*), por ensayo inmunocromatográfico o por ELISA ^8^. Las IgM pueden detectarse luego de cuatro días de iniciado el cuadro febril, mediante ELISA o pruebas rápidas de flujo lateral ^6^, la presencia de IgG indica una infección secundaria o pasada.

La prueba rápida es una herramienta usada en fase aguda de la infección por dengue. Sin embargo, su sensibilidad diagnóstica podría verse afectada de forma negativa en zonas endémicas, ya que las infecciones secundarias por flavivirus son comunes ^9^. En entornos de bajo nivel socioeconómico y acceso limitado a centros de salud, las pruebas rápidas muchas veces se convierten en la única opción para el diagnóstico del paciente, así mismo toma real importancia en estos escenarios para la identificación de brotes de una manera temprana y oportuna; evitando procedimientos que requieren mayor capacitación, bioseguridad, equipamiento e infraestructura compleja ^9^. Por ello, el presente estudio se centra en poder brindar apoyo a regiones endémicas que no cuentan con equipo o personal capacitado para realizar pruebas diagnósticas sofisticadas al momento de captar al paciente. De esta forma se podrá fortalecer las políticas de salud pública en vigilancia epidemiológica, diagnóstico temprano y tratamiento oportuno.

Por todo lo dicho previamente, el objetivo principal de este estudio fue determinar el rendimiento diagnóstico de una prueba serológica rápida para la detección del antígeno NS1 y los anticuerpos IgM e IgG contra el virus del dengue a partir de muestras de suero de pacientes peruanos con síntomas atribuibles a dengue. Para ello se buscó establecer la sensibilidad y especificidad de la prueba rápida para la detección de NS1, IgM e IgG, teniendo como prueba de referencia ELISA indirecto para cada uno de los tres analitos por separado. En este caso se utilizó la prueba ELISA como referencia, ya que esta prueba nos permitió identificar tanto antígeno como anticuerpos y debido a que es la que se encuentra implementada en las regiones del país.

**MENSAJES CLAVE**

Motivación para realizar el estudio: búsqueda de nuevas alternativas diagnósticas de fácil acceso y manejo eficientes para la detección de la enfermedad causada por el virus del dengue.

Principales hallazgos: buena eficiencia de la prueba rápida evaluada en los primeros días de la enfermedad. Así como su alto poder para discriminar frente a otras enfermedades similares transmitidas por mosquitos como el Zika y Oropuche.

Implicancias: podría aplicarse como una prueba de tamizaje en regiones endémicas que no cuentan con equipo o personal capacitado para realizar pruebas diagnósticas sofisticadas y/o complejas al momento de captar al paciente. Fortaleciendo las políticas de salud pública en vigilancia epidemiológica, diagnóstico temprano y tratamiento oportuno.

## MATERIALES Y MÉTODOS

El presente estudio es observacional y de tipo prueba diagnóstica, el cual utilizó muestras de suero recolectado de pacientes con síntomas atribuibles a dengue durante los años 2019-2020, en el marco del Programa de Vigilancia Epidemiológica y que fueron almacenadas a -80 °C en el banco de muestras del Instituto de Investigación Nutricional. Estas muestras cuentan con resultado positivo o negativo a infección por el virus del dengue mediante la prueba Euroimmun ELISA, para los analitos NS1, IgM e IgG por separado. Todos los procesos involucrados en la evaluación de la prueba estudiada fueron llevados a cabo de manera ciega por parte de los analistas, así mismo para la redacción del presente manuscrito se tomó en consideración la guía STARD para estudios de prueba diagnóstica.

Para el cálculo de la muestra se tomó como referencia un estudio realizado por Vásquez *et al*. en el 2012 ^10^, el cual indica que la prueba analizada en ese estudio llegó a una sensibilidad general de 93,8% y una especificidad de 95,8%. Tomando en consideración una precisión del 5,0% se obtuvo un tamaño muestral mínimo de 152, de las cuales 90 serán positivas y 62 serán negativas mediante la prueba ELISA. El presente estudio analizó un total de 286 muestras de suero, de las cuales 203 fueron positivas y 83 negativas mediante la prueba ELISA.

Se determinó el rendimiento diagnóstico, a través del cálculo de los parámetros de sensibilidad, especificidad, valor predictivo positivo y negativo, así como coeficiente Kappa de Cohen y la razón de verosimilitud o *likelihood ratio* (LR) de una prueba rápida que detecta los analitos NS1, IgM e IgG contra el virus dengue denominada SD Dengue Duo-Inyecta en comparación con los resultados obtenidos mediante la prueba Euroimmun ELISA. Adicionalmente, una de las variables evaluadas fue el periodo sintomático, el cual fue medido desde el inicio de los síntomas atribuibles al virus dengue, el cual fue dividido de la siguiente manera: 1-3 días, 4-7 días y 8-14 días. En específico, NS1 aparece en los primeros tres días y desaparece en el día 9, IgM aparece luego de 3 a 5 días de iniciados los síntomas y persiste por aproximadamente dos semanas. Por último, IgG aparece desde el día 10, cuando la infección es primaria; sin embargo, si es secundaria, podría detectarse incluso en el día 4 y puede permanecer elevado por varios meses ^5^. También se evaluaron otras variables, así como edad y sexo.

Finalmente, como medida adicional, dentro de las muestras que fueron negativas se incluyeron 42 muestras que mediante prueba molecular dieron resultado positivo para el virus Oropuche (22), para el virus Zika ^8^ y para Chikungunya ^12^, con la finalidad de evaluar reacción cruzada. Todos los análisis estadísticos fueron realizados con el programa Stata 16.0 (College Station, Tx) y el programa OpenEpi versión 3.

### Prueba rápida SD Dengue DUO®(INYECTA)

La prueba rápida SD Dengue Duo ® (producto 11FK45-P-2, lote 11DDC007A, STANDARD DIAGNOSTIC, INC. Germany) es una prueba inmunocromatográfica que detecta el antígeno NS1 y anticuerpos IgM/IgG en muestra sangre del paciente ^11^. La prueba rápida tiene dos compartimentos, uno para NS1 y el otro para IgM/IgG con una línea de control que siempre debe marcarse para garantizar la validez del resultado. El compartimento izquierdo es para antígeno NS1 donde se agregan tres gotas de suero, y el compartimento derecho es IgM/IgG donde se agregan 10 µl de suero y cuatro gotas de diluyente. Los resultados se obtienen en 20 min, en el caso de NS1 se considera positivo si hay dos líneas marcadas, en el caso de IgM se considera positiva si se marcan la línea de control y la que indica IgM, y en el caso de IgG es positivo cuando se marca la línea de control y la línea de IgG. Todo el procedimiento se realizó conforme lo estipula el fabricante ^12^.

### Prueba ELISA indirecta

Para procesar la muestra mediante esta metodología se necesita un mínimo de sangre de 2 ml. Una vez extraída la muestra se conserva en 2-8 °C hasta enviarla al laboratorio y los resultados son obtenidos en un máximo de tres días.

La presencia del antígeno DENV NS1 y de los anticuerpos IgM/IgG se detectaron usando las pruebas Euroimmun ELISA (Euroimmun AG,Lübeck, Germany) siguiendo las indicaciones del fabricante. Para cada caso se utilizó: ELISA DENV NS1 (lote E211020DU, cat EQ 266a-9601-1); ELISA DENGUE IgM (lote E211202CA, cat EI 266a-9601-1M) y ELISA DENGUE IgG (lote E210312AF, cat EI 266a-9601-1G).

### Aspectos éticos

Se solicitó la autorización del comité de ética de la Universidad Peruana de Ciencias Aplicadas para la realización de este proyecto. Luego de ser aceptada con documento FCS-CEI/281-08-20, se procedió a solicitar la base de datos realizada por el Instituto de Investigación Nutricional. 

Este estudio no implicó riesgos para el paciente, ya que los investigadores solo recolectaron información de las muestras obtenidas por el Programa de Vigilancia Epidemiológica y procesadas por el Instituto Nacional de Nutrición. Además, toda la información recolectada es confidencial y recibió una doble codificación a la cual solo tienen acceso los investigadores del presente estudio. Finalmente, este proyecto de investigación no necesitó consentimiento informado, ya que no hubo un acercamiento al participante puesto que se analizaron los resultados de las pruebas realizadas a las muestras ya recolectadas y procesadas.

## RESULTADOS

Se evaluaron 286 muestras para los analitos NS1, IgM e IgG, mediante la nueva prueba rápida SD Dengue DUO-Inyecta y la prueba de referencia Euroimmun ELISA. Globalmente, se obtuvo que los porcentajes de positividad para NS1, IgM e IgG mediante la prueba rápida fue de 24,5% (70), 13,0% (37) y 52,5% (150) y para la prueba de referencia de 34,0% (97), 19,0% (54) y 54,5% (156), respectivamente. Adicionalmente, el 60,0% de los pacientes se encontraban entre el día 1 y 3, desde que iniciaron los síntomas (tabla 1).

**Tabla 1:** Características clínico-demográficas y resultados de los pacientes con sintomatología atribuible a dengue ingresados al estudio.

Variables	n	%
Sexo		
Masculino	128	55,2
Femenino	158	44,8
Edad (años)	32,8 (2-80) ^a^	
Periodo sintomático		
1-3 días	172	60,2
4-7 días	95	33,2
8-14 días	11	3,8
≥15 días	8	2,8
ELISA IgM		
Positivo	54	19,0
Negativo	232	81,0
ELISA IgG		
Positivo	156	54,5
Negativo	130	45,5
ELISA NS1		
Positivo	97	34,0
Negativo	189	66,0
SD dengue IgM		
Positivo	37	13,0
Negativo	249	87,0
SD dengue IgG		
Positivo	150	52,5
Negativo	136	47,5
SD dengue NS1		
Positivo	70	24,5
Negativo	216	75,5

a Mediana, rango de edad.

IgM: inmunoglobulina M, IgG: inmunoglobulina G, NS1: proteína no estructural, ELISA: ensayo por inmunoadsorción ligado a enzimas

En adición, en la tabla 2 se comparan los resultados obtenidos mediante la prueba rápida SD Dengue DUO-Inyecta y la prueba Euroimmun ELISA para detectar los analitos NS1, IgM e IgG a partir de muestras de suero, encontrándose resultados falsos positivos y negativos para los tres analitos a excepción de lo encontrado para el analito IgM donde no hubo ningún falso positivo. Asimismo, en la tabla 3 se presenta el desempeño de la prueba rápida para la detección de los tres analitos. La sensibilidad fue alrededor del 68,0% para NS1 e IgM y fue un 20,0% más para IgG; en el caso de la especificidad y el valor predictivo positivo de la prueba rápida en general se ubicaron por encima del 87,0% o en valores superiores. Los valores predictivos negativos fueron 83,0% o superiores. Los coeficientes κ de todas las comparaciones se ubicaron en 0,69 o más, mejorando para la detección de IgM que fue de 0,78. Además, la razón de verosimilitud o likelihood ratio (LR) positivo para el antígeno NS1 y para el anticuerpo IgM fueron mayor a 10, así como tuvo un valor de 6,979 para el anticuerpo IgG. De otro lado, el LR negativo fue similar para NS1 (0,33) e IgM (0,31) pero menor para IgG con un valor de 0,16. Adicionalmente, se evidenció que la sensibilidad de la prueba rápida aumenta para los tres analitos durante los tres primeros días de síntomas, sin embargo, al transcurrir los días la sensibilidad disminuye (tabla 4).

**Tabla 2:** Comparación de los resultados obtenidos para NS1, IgM e IgG mediante la prueba rápida SD Dengue DUO-Inyecta y Euroimmun ELISA a partir de muestras de suero de pacientes con sintomatología atribuible a dengue.

Prueba rápida SD Dengue Duo-*Inyecta *Positivo		Euroimmun ELISA		
		Positivo	Negativo	Total
NS1	Positivo	65	5	70
	Negativo	32	184	216
	Total	97	189	286
IgM	Positivo	37	0	37
	Negativo	17	232	249
	Total	54	232	286
IgG	Positivo	134	16	150
	Negativo	22	114	136
	Total	156	130	286

IgM: inmunoglobulina M, IgG: inmunoglobulina G, NS1: proteína no estructural

ELISA: ensayo por inmunoadsorción ligado a enzimas

**Tabla 3:** Rendimiento diagnóstico de la prueba rápida SD Dengue DUO-Inyecta en comparación con la prueba Euroimmun ELISA para la detección de NS1, IgM, IgG en muestras de suero de pacientes con sintomatología atribuible a Dengue.

	NS1 (IC 95%)	IgM (IC 95%)	IgG (IC 95%)
% de sensibilidad	67,0 (57,2-75,6)	68,5 (55,3 - 79,3)	85,9 (79,5 - 90,5)
% de especificidad	97,4 (94,0 - 99,0)	100 (98,4 - 100,0)	87,7 (81,0 - 92,3)
% de VPP	92,9 (84,3 - 96,9)	100,0 (90,6 - 100)	89,3 (83,4 - 93,3)
% de VPN	85,2 (79,8 - 89,3)	93,2 (89,3 - 95,7)	83,8 (76,7 - 89,1)
Kappa de Cohen	0,69 (0,57 - 0.80)	0,78 (0,67 - 0,89)	0,73 (0,62 - 0,85)
LR positivo	25,33	68,52 ^a^	6,979
LR negativo	0,33	0,31	0,16

NS1: proteína no estructural, IgM: inmunoglobulina M, IgG: inmunoglobulina G, VPP: valor predictivo positivo, VPN: valor predictivo negativo, IC 95%: intervalo de confianza al 95%, LR: likelihood ratio

a Para hallar LR+ se usó una especificidad del 99%.

**Tabla 4:** Sensibilidad de la prueba rápida SD Dengue DUO-Inyecta en comparación con la prueba Euroimmun ELISA para la detección de NS1, IgM, IgG según el número de días después del inicio de la sintomatología.

Días después del inicio de síntomas			
SD Dengue DUO-Inyecta	1-3 días (IC 95%)	4-7 día (IC 95%)	8-14 días (IC 95%)
NS1	75,3% (64,4 - 83,8)	61,9% (40,9 - 79,3)	50,0% (9,5 - 90,5)
IgM	80,8% (62,1 - 91,5)	57,7% (39,0 - 74,5)	100% (20,7 - 100,0)
IgG	99,0% (94,5 - 99,9)	91,5% (80,1 - 96,7)	85,7% (48,7 - 97,4)

IgM: inmunoglobulina M, IgG: inmunoglobulina G, NS1: proteína no estructural 1, IC 95%: intervalo de confianza al 95%

Finalmente, se evaluó la reacción cruzada (especificidad analítica) de la prueba rápida SD dengue DUO-Inyecta con diferentes virus como Oropuche, Zika y Chikungunya, obteniéndose un resultado para este parámetro del 100,0%, no encontrándose reacción cruzada de la prueba rápida evaluada con los virus seleccionados.

## DISCUSIÓN

La prueba rápida serológica SD Dengue Duo-Inyecta muestra una sensibilidad por encima del 67,0% y una especificidad por encima del 97,0% para NS1 y para IgM, incrementándose la sensibilidad hasta el 75,0% para el caso de NS1 cuando se evaluaron en los tres primeros días de inicio de síntomas. Estos resultados también fueron encontrados en el estudio publicado por Chung-Hao *et al*. ^9^, en donde encontraron resultados muy similares. Asimismo, se enfatiza que una de las ventajas en el uso de esta prueba es que permite detectar tempranamente la enfermedad y sobre todo en su fase aguda.

Diferentes estudios han evaluado pruebas rápidas utilizadas para la detección de analitos contra dengue, estas en su mayoría han sido comparadas con pruebas diagnósticas más complejas (requieren infraestructuras elaboradas, personal altamente capacitado y equipamiento especializado). Es así, como en el 2012 se evaluó la prueba diagnóstica SD Bioline Dengue Duo en comparación con una prueba diagnóstica estándar denominado Platelia NS1, obteniéndose una sensibilidad de 57,8% y una especificidad de 98,0% ^10^. Así mismo, otro estudio evalúo la prueba rápida Dengue NS1 Ag STRIP dando como resultado final una sensibilidad del 63,8% en 392 pacientes confirmados para dengue ^9^. Adicionalmente, otro estudio analizó la efectividad diagnóstica de la «prueba rápida NS1 e IgM de dengue» en Colombia, en un periodo pre-zika del 2019, reportando una sensibilidad diagnóstica clínica de 61,4% ^11^. Por otro lado, un trabajo realizado en el Perú, en el 2013, en Lambayeque, que también analizó la efectividad diagnóstica de la prueba rápida SD Bioline Dengue Duo en comparación con la prueba de ELISA obtuvo una sensibilidad del 88,5% ^13^.

En el presente estudio se obtuvo una sensibilidad de 67,0% y 68,5% y una especificidad de 97,4% y 100,0%, para NS1 e IgM, respectivamente. Los resultados de sensibilidad y especificidad obtenidos para IgG se encuentran alrededor del 86,0%, y esto puede deberse a la presencia de una importante proporción de infecciones secundarias, por lo cual se debe tener mucha cautela al momento de interpretar estos resultados. Estos datos son similares a los estudios publicados por Clemen *et al*. en el 2019 ^11^ así como el estudio que evaluó la prueba rápida Dengue NS1 Ag STRIP ^9^. En adición, la sensibilidad obtenida para IgM y NS1 en nuestro estudio fue mayor a lo encontrado en otros artículos que también evaluaron el rendimiento diagnóstico de diferentes pruebas rápidas, esto podría deberse a que no se analizó los diferentes serotipos de DENV, ya que si predomina el serotipo DENV 2 y 4 se altera la sensibilidad del antígeno NS1. Sin embargo, los resultados de la sensibilidad fueron mejores en el trabajo realizado en el Perú, en el 2013 ^13^, quizás esta mejora de la sensibilidad en algunas pruebas evaluadas pueda deberse a que no se tuvo en cuenta el periodo sintomático de la enfermedad, aumentando el valor de este parámetro. Asimismo, otros estudios evaluaron la sensibilidad y especificidad de diferentes pruebas rápidas, obteniendo resultados similares ^10^ ^,^ ^14^ ^-^ ^15^.

Por otro lado, en el 2019 ^11^ se buscó comparar la eficacia diagnóstica de la prueba rápida SD dengue DUO con el PCR tiempo real (*gold standard*). Aquel estudio tuvo como resultado una sensibilidad de 85,4% y una especificidad de 94,5% para la prueba rápida, mientras que el presente estudio demostró una menor sensibilidad para NS1 e IgM y una especificidad similar para NS1 e IgM, pero menor para IgG. Esta diferencia hallada entre ambos estudios puede ser causada por la prueba de referencia utilizada, ya que la primera evaluación se realizó con base en PCR y la nuestra con base en ELISA indirecto. Esto último con la idea de poder buscar el reemplazo de la prueba que masivamente está implementada por el MINSA en las regiones del Perú, que es el ELISA.

En esta investigación, más del 50,0% de la población que ha ingresado al estudio son positivos para anticuerpos IgG, lo que sugiere una infección secundaria, esto además ocasiona un factor independiente para un resultado negativo de NS1. Se ha pensado que la causa sería la similitud entre la cinética de NS1-IgG específica y la IgG de DENV que ocasiona la formación de complejos inmunes por la unión del antígeno NS1 y NS1-IgG específica que es un anticuerpo que aparece a los ocho días en una infección primaria, pero en una secundaria aparece en el primer día, lo cual reduce su sensibilidad ^16^. Asimismo, al evaluar los valores predictivos para cada uno de los analitos estudiados es importante tener en cuenta que los resultados deben ser tomados con cuidado, ya que este parámetro depende de la prevalencia de la enfermedad, por lo que estos resultados solo pueden ser extrapolados para lugares en donde las prevalencias son parecidas y tomando en cuenta el analito determinado. 

Adicionalmente, el coeficiente *Kappa* de Cohen indica que la posibilidad de tener un resultado negativo por la prueba rápida cuando es positivo por ELISA es baja. Del mismo modo, la posibilidad de tener un resultado positivo por la prueba rápida cuando es negativo por ELISA es mayor de la mencionada anteriormente. Datos similares a los obtenidos por Sáenz Bolaños *et at*. en la evaluación de prueba rápida contra el MAC-ELISA en Costa Rica ^17^ y esto se puede deber a la similitud del trabajo con el nuestro y la utilidad de la prueba rápida en la fase aguda de la enfermedad en la detección de anticuerpos IgM.

De la misma manera, en lo que respecta al LR obtenido en el presente estudio, NS1 e IgM mostraron un LR + altamente relevante, ya que es 25,33 y 68,52 veces más verosímil que un paciente con ELISA positivo obtenga una prueba rápida positiva a dengue que un paciente con ELISA negativa tenga el mismo resultado, también se halló una buena utilidad en IgG (6,79). En cuanto a LR negativo se halló un valor útil en IgG (0,16) y regular en los otros analitos (0,31) por lo que se afirma que los resultados negativos de la prueba rápida ocurren 0,31 veces en pacientes con ELISA positivo con respecto cada muestra con ELISA negativo. Adicionalmente, la interpretación de estos resultados tiene un rol importante, sobre todo cuando se define la prevalencia de la enfermedad, ya que considerando diferentes escenarios de prevalencia de la enfermedad (bajo, medio o alto) y con los resultados LR obtenidos mediante la prueba se podría valorar mejor la interpretación clínica que este parámetro pueda otorgar ^18^.

Adicionalmente se halló que el rendimiento diagnóstico de la prueba rápida cambia de acuerdo con el periodo sintomático del paciente y el tipo de analito. La detección de IgM en nuestro estudio llegó a una sensibilidad de 80,77% en los tres primeros días de enfermedad, desde la aparición de los síntomas. Esto también ocurre con el antígeno NS1 que llegó a su mayor punto de sensibilidad en los primeros tres días, un 75,3%. Lo cual ratifica su utilidad en la fase aguda de la enfermedad y que al pasar los días esta sensibilidad va disminuyendo. Sin embargo, la alta sensibilidad en los primeros días para la detección de IgG es un indicador de reinfección por el virus. 

Por otro lado, al evaluar la especificidad analítica de la prueba rápida SD Dengue Duo-Inyecta con respecto a otros Arbovirus como lo son el Zika, Chikinguya y Oropuche. La prueba rápida demostró una especificidad del 100,0% para dengue, lo que indica que no hay reacción cruzada con otros arbovirus, validando la utilidad de esta prueba rápida en zonas endémicas para estas patologías. No se evaluó reacción cruzada como el virus Mayaro pese a que se ha descrito un aumento de su distribución en Perú ^19^.

Finalmente, dentro de los principales hallazgos de la presente investigación, se resalta la eficiencia de la prueba rápida en los primeros días de la enfermedad, así como su capacidad para distinguir la enfermedad de dengue con tipos similares como el Zika u Oropuche, que son enfermedades también transmitidas por mosquitos. Adicionalmente, entre las limitaciones encontradas se mencionan que la información clínico-epidemiológica fue escasa y esto debido a que las muestras ya estaban recolectadas para un objetivo diferente y se encontraban almacenadas en el banco de muestras del laboratorio. Asimismo, el no usar la prueba de PCR que es considerada la prueba *gold standard* en el diagnóstico de dengue, limitó evaluar la sensibilidad y especificidad de la prueba rápida con respecto a la prueba molecular, sin embargo, el objetivo también estuvo dirigido a poder reemplazar la prueba ELISA que es la que se encuentra implementada en las diferentes regiones del país. También es importante mencionar que el periodo sintomático fue brindado por el paciente pudiendo haberse producido un sesgo de memoria al dar incorrectamente la fecha de inicio de síntomas y por último el no conocer el serotipo de dengue o si la infección fue primaria o secundaria podría alterar el rendimiento de la prueba rápida evaluada.

En conclusión, el rendimiento diagnóstico de la prueba serológica rápida SD Dengue Duo-Inyecta para la detección del antígeno NS1 y anticuerpos IgM e IgG indica que es un método aceptable para el diagnóstico temprano de dengue. Asimismo, en pacientes que cuentan con más de siete días de inicio de síntomas, los resultados deben ser tomados con cautela, ya que la sensibilidad de la prueba se puede ver afectada.

Se recomienda su implementación en establecimientos de atención primaria en zonas endémicas para un diagnóstico rápido y oportuno, reduciendo la transmisión y complicación de la enfermedad, asimismo, facilitar la detección temprana de posibles brotes. No se debe olvidar que la prueba rápida brinda un diagnóstico presuntivo de utilidad sin embargo no da un diagnóstico definitivo por lo cual también se debe realizar otra prueba como ELISA o PCR, para lograr la respectiva confirmación.
